# Flare-Up Diverticulitis in the Terminal Ileum in Short Interval after Conservative Therapy: Report of a Case

**DOI:** 10.1155/2016/8162797

**Published:** 2016-12-20

**Authors:** Kensuke Nakatani, Takaharu Kato, Shinichiro Okada, Risa Matsumoto, Kazuhiro Nishida, Hiroyasu Komuro, Maki Iida, Shiro Tsujimoto, Toshiyuki Suganuma

**Affiliations:** ^1^Department of Surgery, Yokosuka General Hospital Uwamachi, 2-36 Uwamachi Yokosuka City, Kanagawa 238-8567, Japan; ^2^Department of Surgery, Saitama Medical Center, Jichi Medical University, 1-847 Amanuma-cho, Omiya-ku, Saitama 330-8503, Japan; ^3^Department of Pathology, Yokosuka General Hospital Uwamachi, 2-36 Uwamachi, Yokosuka, Kanagawa 238-8567, Japan

## Abstract

Diverticulitis in the terminal ileum is uncommon. Past reports suggested that conservative therapy may be feasible to treat terminal ileum diverticulitis without perforation; however, there is no consensus on the therapeutic strategy for small bowel diverticulitis. We present a 37-year-old man who was referred to our hospital for sudden onset of abdominal pain and nausea. He was diagnosed with diverticulitis in the terminal ileum by computed tomography (CT). Tazobactam/piperacillin hydrate (18 g/day) was administered. The antibiotic treatment was maintained for 7 days, and the symptoms disappeared after the treatment. Thirty-eight days after antibiotic therapy, he noticed severe abdominal pain again. He was diagnosed with diverticulitis in terminal ileum which was flare-up of inflammation. He was given antibiotic therapy again. Nine days after antibiotic therapy, laparoscopy assisted right hemicolectomy and resection of 20 cm of terminal ileum were performed. Histopathology report confirmed multiple ileal diverticulitis. He was discharged from our hospital 12 days after the surgery. Colonoscopy was performed two months after the surgery and it revealed no finding suggesting inflammatory bowel disease. Surgical treatment should be taken into account as a potential treatment option to manage the diverticulitis in the terminal ileum even though it is not perforated.

## 1. Introduction

Diverticulitis in the terminal ileum is an uncommon entity except for Meckel's diverticulum. Pathologically, the diverticulosis in small bowel is characterized by herniation of the mucosa and the submucosa through the muscular layer of the bowel wall [1, 2]. Some case reports showed patients who received surgery for perforated diverticulitis in the small intestine [3–7]. But the standardized therapy has not been established in the patient with diverticulitis which was not perforated. We present a case of diverticulitis in the terminal ileum in a middle-aged man which was flare-up of inflammation in short interval after antibiotic therapy and needed surgical treatment.

## 2. Case Presentation

**Figure 1:**
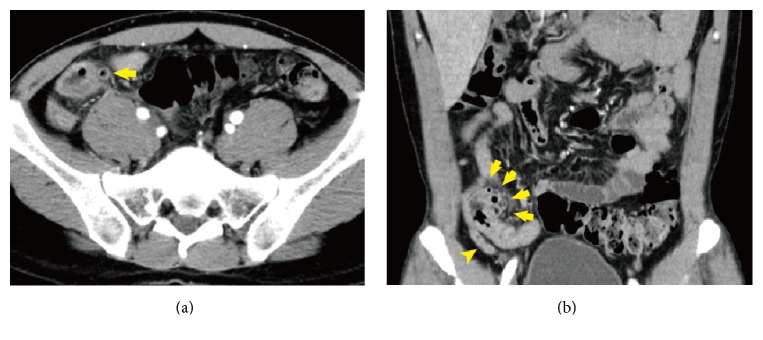
Computed tomography revealed diverticula sequentially located in the wall thickening terminal ileum (arrows); surrounding abdominal fat was developing high density suggesting inflammation. The appendix was not swollen (arrow head).

**Figure 2:**
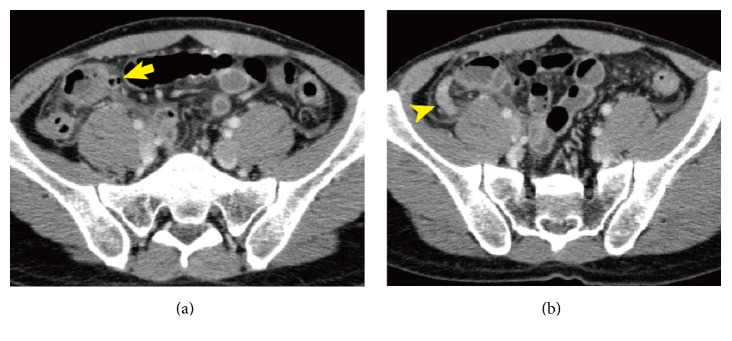
Computed tomography revealed diverticula in the terminal ileum (arrow) and swollen appendix with 10 mm in the diameter (arrow head).

**Figure 3:**
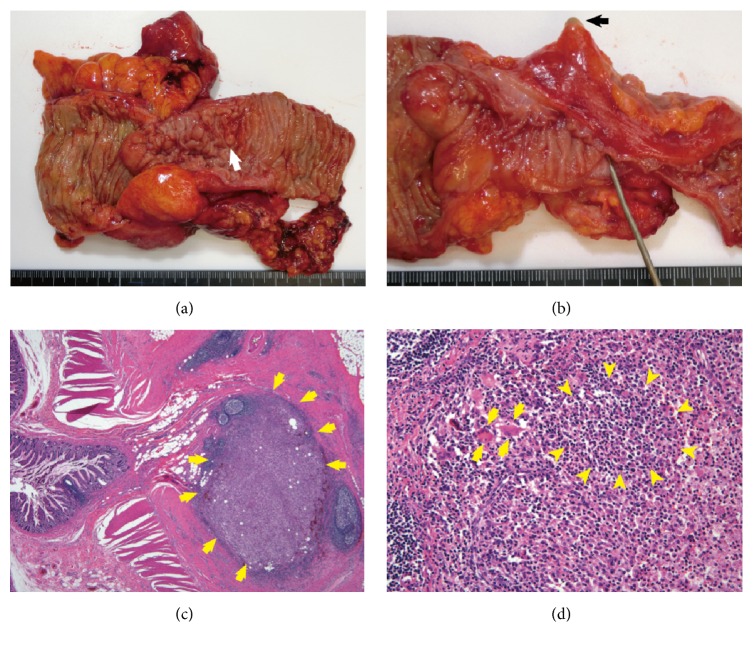
Surgically resected specimen revealed diverticula on the mesenteric side in the terminal ileum ((a) and (b)). Microscopically, the nodular areas correspond to points of mucosal invagination into the surrounding muscular layer, creating diverticula (c). There is inflammatory granuloma that consisted of foreign body giant cells (arrows in (d)) and foam cells (arrow heads in (d)) in the invaginated area.

A 37-year-old man woke up at midnight with severe abdominal pain and nausea. The patient consulted local clinic and was diagnosed with acute abdomen. He was referred to Yokosuka General Hospital Uwamachi for further examination and treatment. On physical examination, his blood pressure was 118/67 mmHg with a pulse rate of 103 beats and respiratory rate of 18 per minute and body temperature of 38.2 degrees C. He had rebound tenderness in his right lower quadrant. Laboratory analysis showed a C-reactive protein level of 2.6 mg/L, and white blood cell count was 20,700/*μ*L. Computed tomography (CT) revealed sequential diverticula in the wall thickening terminal ileum with high density of surrounding fat (arrows in Figures 1(a) and 1(b)) and appendix without swelling (Figure 1(b) arrow head) and also multiple uncomplicated diverticula in the ascending colon separated from the panniculitis lesion were detected. He was diagnosed with diverticulitis in the terminal ileum. Tazobactam/piperacillin hydrate (18 g/day) was administered. Antibiotic therapy was maintained for 7 days and the symptoms disappeared. While the peak level was 12.89 mg/dL, C-reactive protein level was deceased to 0.92 mg/dL after the treatment. Following this treatment, the patient was started on potassium clavulanate and amoxicillin hydrate (1500 mg/day) and discharged from our hospital 10 days after the admission. Nineteen days after his discharge, he had been seen in the follow-up consultation without any inflammatory findings. Three weeks after his last consultation, 38 days after antibiotic therapy was finished, he noticed severe abdominal pain and nausea again and was carried to the local hospital. He was diagnosed with diverticulitis in the terminal ileum and acute appendicitis by CT examination performed in the local hospital. He was referred to our hospital for possible surgery. On physical examination, his blood pressure was 111/70 mmHg with a pulse rate of 90 beats and respiratory rate of 20 per minute and body temperature of 37.2 degrees C. He had rebound tenderness in his right lower quadrant. Laboratory analysis showed a C-reactive protein level of 1.2 mg/L, and white blood cell count was 13,600/*μ*L. Abdominal enhanced CT performed at previous hospital which showed diverticula in the terminal ileum with high density of surrounding fat (Figure 2(a) arrows) and appendix with 10 mm swelling (Figure 2(b) arrow head). On the whole, CT findings were similar with those of primary diverticulitis. He was diagnosed with diverticulitis in the terminal ileum which was flare-up of inflammation and acute appendicitis. Tazobactam/piperacillin hydrate (18 g/day) was administered again. Since the diverticulitis was flare-up of inflammation in short interval after conservative therapy, we decided to perform surgery. Nine days after antibiotic therapy, laparoscopy assisted right hemicolectomy and resection of 20 cm of terminal ileum were performed. Meckel's diverticulum was not found in the ileum. The resected specimen revealed diverticulitis in the terminal ileum on the mesentery side (Figures 3(a) and 3(b), arrows). On microscopic evaluation, the nodular areas correspond to points of mucosal invagination into the surrounding muscular layer, creating diverticula (Figure 3(c)). There was inflammatory granuloma that consisted of foreign body giant cells (Figure 3(d) arrows) and foam cells (Figure 3(d) arrow heads) in the invagination area. The swelling appendix did not have any inflamed mucosa, however, it showed serosal marked inflammation with hemorrhage, which was associated with ileal diverticulitis. He was discharged 12 days after the surgery without any complication. Two months after the surgery, colonoscopy was performed that showed no finding suggesting inflammatory bowel disease or other diseases.

## 3. Discussion

We present a case of diverticulitis in the terminal ileum which was flare-up of inflammation in 38 days after conservative therapy and needed surgical treatment. Diverticulitis in the terminal ileum is uncommon [8–10]. Diverticular disease is more common in the proximal jejunum (75%), followed by the distal jejunum (20%) and the ileum (5%) [11]. As previous reports had showed that associated colonic diverticulosis is frequently found in the patients with small bowel diverticulosis [12, 13], the present patient also developed coexistent diverticula in the ascending colon. The present patient was not elderly, but most patients were in the sixth and seventh decade of life in the previous reports [14–17]. Barton et al. [18] reported a patient with familial jejunoileal diverticulitis, but the present patient does not have familial history of diverticulitis in the small intestine. Although the etiology of small bowel diverticula has been unknown, this condition is believed to develop from a combination of intestinal motility disorders, focal weakness of the muscle, and high segmental intraluminal pressure [6, 19]. The majority of small bowel diverticula are asymptomatic, but a wide range of less common complications has been reported, including abscess formation, chronic abdominal pain, malabsorption, anemia, volvulus, biliary tract disease, and enterolith formation [20]. Compared with duodenal diverticula, small bowel diverticula were nearly 4 times more likely to develop complications and nearly 18 times more likely to perforate and develop abscesses [20]. Several reports suggested that conservative management may be feasible to treat terminal ileum diverticulitis without perforation [21–23]; however there is no consensus on the therapeutic strategy of the symptomatic small bowel diverticulitis. In the previous reports, small bowel diverticula have a risk of perforation [4, 16, 24, 25]. Surgical intervention might be one option for diverticulitis without perforation. To prevent development of acute diverticulitis, high-fiber diet is commonly recommended to optimize their bowel movement for the patients with colon diverticulosis [26–28]. But recent studies have not supported recommendation of high-fiber diet [29, 30]. The patient does not have an unbalanced diet but has average Japanese dietary habits.

In conclusion, we present a case of diverticulitis in the terminal ileum in a healthy middle-aged man which was flare-up of inflammation in short interval after conservative therapy and needed surgical intervention. We should follow up with the patients cautiously who finished conservative therapy for diverticulitis without perforation in the terminal ileum not to overlook the flare-up diverticulitis. Surgical treatment might be considered as a potential treatment option to manage the small bowel diverticulitis.
